# Huggable Communication Medium Maintains Level of Trust during Conversation Game

**DOI:** 10.3389/fpsyg.2017.01862

**Published:** 2017-10-25

**Authors:** Hideyuki Takahashi, Midori Ban, Hirotaka Osawa, Junya Nakanishi, Hidenobu Sumioka, Hiroshi Ishiguro

**Affiliations:** ^1^Graduate School of Engineering Science, Osaka University, Osaka, Japan; ^2^Exploratory Research for Advanced Technology (ERATO), Japan Science and Technology Agency, Osaka, Japan; ^3^Faculty of Psychology, Doshisha University, Kyoto, Japan; ^4^Human-Agent Interaction Laboratory, University of Tsukuba, Tsukuba, Japan; ^5^Advanced Telecommunications Research Institute International, Kyoto, Japan

**Keywords:** remote communication device, huggable communication medium (*Hugvie*), hugging experience, trust, conversation-based game (*Werewolf*)

## Abstract

There have been several attempts in recent years to develop a remote communication device using sensory modalities other than speech that would induce a user’s positive experience with his/her conversation partner. Specifically, *Hugvie* is a human-shaped pillow as well as a remote communication device enabling users to combine a hugging experience with telecommunication to improve the quality of remote communication. The present research is based on the hypothesis that using *Hugvie* maintains users’ level of trust toward their conversation partners in situations prone to suspicion. The level of trust felt toward other remote game players was compared between participants using *Hugvie* and those using a basic communication device while playing a modified version of *Werewolf*, a conversation-based game, designed to evaluate trust. Although there are always winners and losers in the regular version of *Werewolf*, the rules were modified to generate a possible scenario in which no enemy was present among the players and all players would win if they trusted each other. We examined the effect of using *Hugvie* while playing *Werewolf* on players’ level of trust toward each other and our results demonstrated that in those using *Hugvie*, the level of trust toward other players was maintained.

## Introduction

The proliferation of various remote communication devices such as mobile phones in recent years has turned them into an essential part of our daily lives. To induce the positive experience in remote communication beyond the role of merely information exchanges, efforts have been made to explore the use of various additional sensory modalities in communication device such as visual information in video communication (Isaacs and Tang, 1994; Finn et al., 1997; De Greef and Ijsselsteijn, 2001).

In addition to visual information, the uses of tactile stimuli in communication devices to contribute the positive experience of users in remote conversation has recently gathered a great deal of attention. From a psychological perspective, it has been suggested that tactile sensations play an important role in building rapport in interpersonal communication (Gallace and Spence, 2010; Tatsukawa et al., 2016). Thus, there have been attempts to integrate tactile functions into various media (Chang et al., 2002; Prattichizzo et al., 2010). For instance, it has been reported that the level of mutual closeness experienced by participants during a video chat increased when they had artificial contact in the form of a handshake with a robotic hand (Nakanishi et al., 2014). Furthermore, an attempt has been made to attach a device to a smartphone to enable users to experience an artificial sensation of kissing during remote communication (Samani et al., 2012). However, it should be noted that most studies evaluating the serviceability of these devices were conducted using controlled, predetermined conversations. In other words, very few studies have explored the psychological and behavioral effects of remote communication devices with tactile sensations using conversational scenes allowing free utterances. Therefore, we posit that while the market explores commercial applications for remote communication devices with tactile functions, serviceability studies using unconstrained communication are necessary.

**FIGURE 1:**
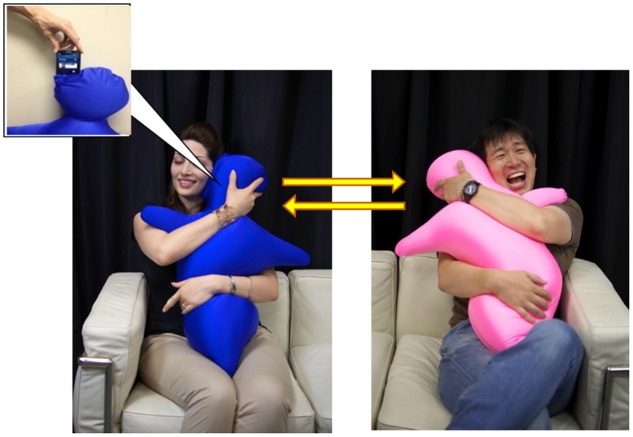
*Hugvie* (huggable communication medium).

*Hugvie* is a human-shaped cushion aimed at providing users with the tactile stimulation of hugging during phone conversations in order to improve positive feelings (e.g., closeness and trust) toward each other (Sumioka et al., 2013, 2014; Yamazaki et al., 2016), and its serviceability has been demonstrated using free-conversation scenes (**Figure 1**). A psychological study reported that conversations with a female while hugging *Hugvie* stimulated male users’ feelings of closeness toward her (Kuwamura et al., 2014). Furthermore, Sumioka et al. (2013) compared the level of cortisol, a hormone positively correlated with stress levels, between participants using *Hugvie* and those using a basic mobile phone during an unconstrained remote conversation, and showed that the concentration of cortisol in blood and saliva decreased in those using *Hugvie*. This result is extremely compelling, as it suggests that *Hugvie* users were more relaxed based on a physiological indicator. These psychological and physiological data have suggested that using *Hugvie* has positive effects on mutual closeness during a remote conversation. In addition to these findings, we expect that *Hugvie* has a strong effect in the formation of various social attitudes. Especially, we hypothesize that *Hugvie* enhances the trust of users, because previous psychological findings have suggested that the sense of interpersonal touch is strongly linked to the formation of trust (Gallace and Spence, 2010; Walter et al., 2013). If we investigate this hypothesis, a good experimental task that allows free-conversation and enabled to quantify the trust is required.

Conversation games allow free-flowing conversations between players; however, controlled responses according to the game procedure can also be objectively observed. Hence, these games are useful to validate the effects of *Hugvie* on trust during a free conversation, as it possible to quantify controlled responses in the game. The present study utilized a conversation-based game known as *Werewolf*, which was modified to evaluate the level of trust between players. One group of participants used *Hugvie* to play the game with remote players, while the other participant group used a basic communication device. Changes in participants’ level of trust toward other players were compared between the two groups. Collected data involved controlled responses (vote behavior) during the game and responses to a questionnaire. One of the advantages of using this type of conversation-based game was that although participants could talk freely, we could easily gauge their state of mind based on the actions invariably required to play the game. Although there are always winners and losers in the regular version of *Werewolf*, the game rules were modified in the present research to include possible scenarios in which there was no enemy and all players would win if they trusted one another. This modification allowed us to evaluate the potential increase in mutual trust caused by using *Hugvie*.

## Materials and Methods

### Experimental Conditions and Participants

**FIGURE 2:**
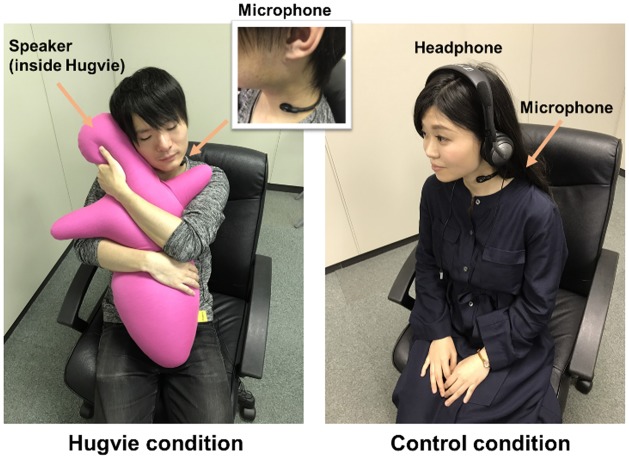
Body posture during experiment in two conditions.

The design of the present research was based on a comparison between two groups of participants, the *Hugvie* and the control group. Those in the *Hugvie* group used *Hugvie* to talk to remote players during the *Werewolf* game, while those in the control group used a basic communication device instead. There were no other significant differences between the two conditions (**Figure 2**, details of body posture during experiment in two conditions).

There were 12 participants in the *Hugvie* group (average age = 20.7 years; three females) and 12 in the control group (average age = 20.5 years; three females). The validity of the sample size was confirmed through *post hoc* analyses of effect size to a certain extent, as our experimental paradigm was novel and there were no previous studies suggesting an appropriate sample size. None of the participants had a history of neurological or psychiatric illness. All participants provided written informed consent prior to the start of the study, which was approved by the Ethics Committee of Doshisha University, Japan. Furthermore, the composition of both participant groups was similar in terms of participants’ relationships with one another and sex. For both groups, members of two of the male teams were friends, while the rest of the teams consisted of strangers. Both female teams were composed of strangers. Furthermore, only participants who had previously played *Werewolf* several times and knew its general rules were recruited into the study.

### Huggable Communication Medium: *Hugvie*

*Hugvie*, a huggable communication medium, is a human-shaped cushion (height: 75 cm; weight: 600 g) designed as a communication device to give users a hugging experience. It is a soft cushion filled with polystyrene microbeads and covered with a material composed of acrylic and rayon, commonly used for blankets. It resembles a person opening his/her arms offering a hug and combines the hugging experience with telecommunication through the insertion of a hands-free mobile phone into a pocket in *Hugvie*’s “head.” Since the phone is in the pocket, users can talk while hugging *Hugvie*, creating the feeling of hugging a distant conversation partner. *Hugvie* does not have any actuators inside it, thereby enabling investigation of the effects of its inactive touch.

To maintain stable audio communication, we used throat microphones (Dharma Tactical Throat Microphone DRMC01P, Sigma A.P.O. System Sales, Co., Ltd.) and 2.4 GHz digital wireless speakers (customized version of DW-05, Azden Corporation). Participants’ vocal input to the microphone was output through all players’ speakers. Therefore, all participants could hear their own and other players’ voices. For participants using *Hugvie*, we placed the speaker inside a pocket in *Hugvie*’s head and asked participants to place the speaker next to their ears to hear players’ voices. Identical microphones and speakers were used for control participants; however, the speakers were worn as headphones (**Figure 2**).

### Modified *Werewolf* Game

*Werewolf* is a conversation-based multi-player game (Hung and Chittaranjan, 2010; Kobayashi et al., 2014). Its regular version, based on free-flowing conversations with a time limit, begins with each player being given an identity (mainly either “villager” or “werewolf”) known only to him/her. The werewolves are meant to hide their identity and participate in conversations while pretending to be villagers. The villagers’ goal is to guess who the werewolves are based on the conversations, while the werewolves attempt to prevent the villagers from uncovering their true identity. After the time for conversation reaches the limit, each player casts a vote guessing who the werewolves are, and the player receiving the most votes is eliminated. If the real werewolves have avoided elimination, they secretly choose one villager to be attacked and he/she is eliminated, which concludes a round. The game continues through a repetition of rounds until either all the werewolves are eliminated (the villagers win) or there is an equal number of villagers and werewolves (the werewolves win). Although the villagers guess the identity of the werewolves based solely on information gathered through conversation, there can also be villagers with special talents. For instance, a “seer” can discover the identity of one person per round, identifying whether he/she is a villager or a werewolf. This type of information may be useful for the villagers; however, a werewolf can also pretend to be a seer because players’ identities are kept secret throughout the game, which may lead to players employing complex strategies. To play *Werewolf*, players are required to have various social skills, including reading people and pretending to be someone other than themselves. Beyond mere entertainment, this aspect of the game has recently garnered much attention from researchers in cognitive science and artificial intelligence as a tool to investigate human communication skills (Hung and Chittaranjan, 2010; Kobayashi et al., 2014).

**FIGURE 3:**
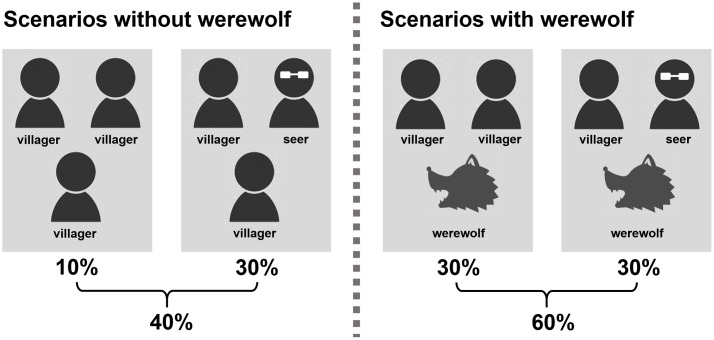
Possible scenarios and probabilities of occurrence.

The present study used a version of *Werewolf* modified to accommodate the purpose of the research. The regular version of the game includes five players or more; however, to facilitate analysis, the modified version included only three. Furthermore, the modified version concluded after a one-round session of free-flowing conversation followed by a vote, and the attack phase by the werewolves was omitted. The most significant change, however, was that a new scenario in which no werewolf was present among the players was created to evaluate the degree of trust. Specifically, for each game, three slots for villagers, one for a seer, and one for a werewolf were set up, which were then randomly assigned to three players. In other words, in terms of possible scenarios, the game could have three villagers (10%), two villagers and a seer (30%), two villagers and a werewolf (30%), or a villager, a seer, and a werewolf (30%). Therefore, the probability that a werewolf was in a team was 60%, and that of having no-werewolf in the team was 40% (**Figure 3**). Moreover, unlike the regular version, the players cast a vote anonymously predicting whether there was a werewolf among them (“No werewolf” votes) before voting to name the werewolf. If all three players voted in favor of the “No werewolf” option, the game was over at that point. Conversely, if at least one player voted that there was a werewolf among them, the game moved on to voting to name the werewolf. However, if in fact there was no werewolf among the players, all villagers lost at that point in the game. Each player voted anonymously which of the other two players he/she thought was the werewolf. A player was eliminated if he/she received two votes, whereas if each player received one vote, no one was eliminated. If the actual werewolf was eliminated during this process, the villagers would win; if a villager was eliminated or there was no elimination, the werewolf would win.

These modifications made it possible to have no werewolf among the players and thus created a situation where players could not be too trusting or suspicious toward each other if they were to increase their chances of winning. The purpose of these changes was to examine, based on their voting patterns, how players learned to trust that other participants were villagers rather than werewolves in an ambiguous setting due to lack of information as to whether a werewolf existed or not. With this modification, it became possible to quantify the level of trust by using the ratio of “No werewolf” votes. This modification was based on the idea that, in many situations, trust involves the intuition to believe other persons without any logic or rational reasons (Schoorman et al., 2007).

### Procedure

The experiment began as three participants gathered in the same experiment room. They were then asked to introduce themselves to one another and were randomly assigned a letter (A to C) to facilitate identification during the conversation. They sat on chairs in three separate corners in the same large room to complete a questionnaire consisting of 16 items using a seven-point Likert scale regarding their impressions, such as trustworthiness and cleverness (see graph labels in **Figure 3** for details), of the other two players. Questionnaire items were chosen *ad hoc* to fit the research purpose. The game began when the light was turned off. At the beginning of the game, participants were instructed not to talk directly with others but to whisper into their remote communication devices. Participants played a total of five rounds (3 min of conversation in each round). The first was a practice round in order for participants to understand the game and rules. After finishing the last round, participants completed the questionnaire again, enabling an evaluation of changes in their impressions. Participants had no prior knowledge regarding the number of rounds to be played in the experiment and were instructed to win the game as often as possible; however, no incentives were paid depending on participants’ winning rate.

Each round followed the same pattern. First, at the beginning of the round, each player was randomly assigned an identity, which was displayed on a tablet (Surface pro) presented by the researcher. Only the identity assigned to the player would be visible on the tablet screen; however, if the player was to be a seer, the identity (villager or werewolf) of a randomly chosen player would also be displayed. Subsequently, using either *Hugvie* or the basic communication device, participants spoke to one another freely in whispering voices for 3 min to decide whether there was a werewolf among them and, if so, who that might be. After 3 min, each participant voted to indicate whether he/she thought that there was a werewolf among them by pressing a button displayed on the tablet touchscreen. If at least one participant guessed correctly that there was a werewolf among them, participants cast a vote to guess who the werewolf was. Throughout this process, each participant’s vote was kept secret from other players during and after a given round. Winners and losers were determined according to the aforementioned rules. An artificial voice then announced one of the three possible outcomes: “the villagers win,” “the werewolf wins,” or “the village is destroyed” (this happened when someone guessed that there was a werewolf among them when there was none).

## Results

Firstly, we verified that identities had been assigned equally in the two experimental groups. In the *Hugvie* group, “villager” identities accounted for 70%, “seer” for 17%, and “werewolf” for 13%. In the control group, “villager” identities were assigned to 62% participants, “seer” to 18%, and “werewolf” to 20%. There was no significant difference in the proportion of identities between the two groups [chi-square test, X^2^(2) = 0.452, *p* = 0.8, Cramer’s *V* = 0.069].

**FIGURE 4:**
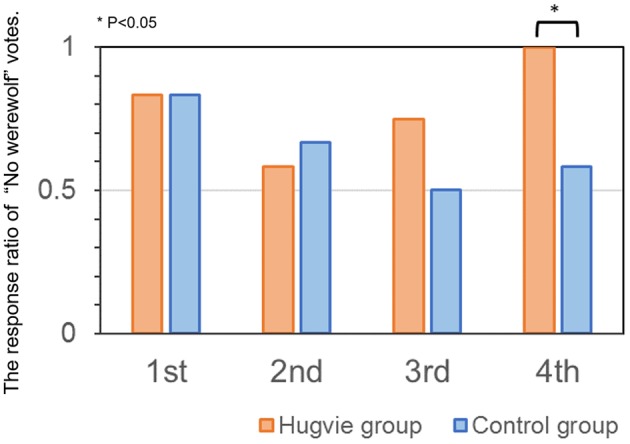
Ratio of “No werewolf” votes for each round and group.

Subsequently, we compared the ratio of “No werewolf” votes between the two groups. Specifically, we calculated the ratio of participants who voted “No werewolf” in each round and group (**Figure 4**). The ratio of “No werewolf” votes was found to be higher in the *Hugvie* group than in the control group only in the 4th round [chi-square test, X^2^(1) = 2.51, *p* = 0.012, φ = 0.32, stochastically significant after the Bonferroni multiple-comparison correction]. Furthermore, we also compared the overall mean ratio of “No werewolf” votes between male and female subjects and we could not find the significant difference between two [independent *t*-test, *t*(22) = -0.801, *p* = 0.43, *d* = 0.38].

**FIGURE 5:**
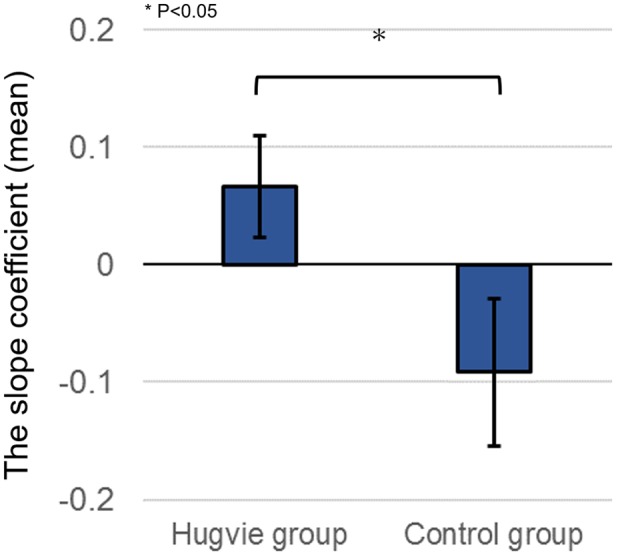
Means of the slope coefficients in the two groups.

Additionally, we examined how the level of trust toward other participants changed during repetitive rounds. The choice of “No werewolf” votes was set as the dependent variable representing participants’ trust behavior in a round, with a value of 1 when a participant voted “No werewolf” in the round and 0 when the participant did not vote it. Subsequently, we estimated a linear regression model per subject predicting the change in the dependent variable according to round number (explanatory variable). The slope coefficient of this regression model indicates how the level of trust changed through repetitive rounds. Positive slope values indicate that the level of trust increases during repetitive rounds while negative slope values mean that it decreases. We compared the means of the slope coefficients between the two groups and found that the value in the *Hugvie* group was significantly higher than that in the control group [**Figure 5**, independent *t*-test, *t*(22) = 2.181, *p* = 0.04, *d* = 0.89]. This suggests that the level of trust toward other participants in the *Hugvie* group was relatively maintained in comparison with that in the control group.

**FIGURE 6:**
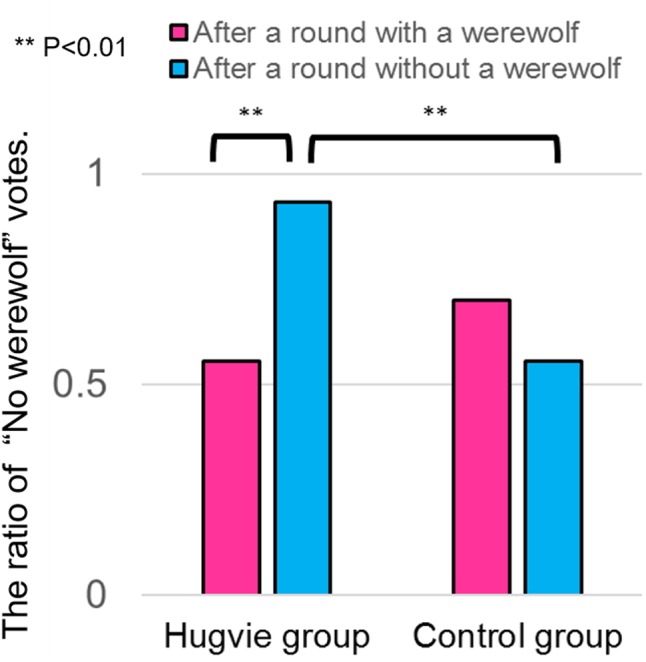
Ratio of “No werewolf” votes after a round with/without a werewolf for each group.

Furthermore, we analyzed how the performance in a previous round affected participants’ trust behavior in the current round and found that the ratio of “No werewolf” votes after a round without a werewolf (i.e., a werewolf did not exist in the prior round) was significantly higher than the ratio of “No werewolf” votes after a round with a werewolf (i.e., a werewolf existed in the prior round) in the *Hugvie* group [chi-square test, X^2^(1) = 7.58, *p* = 0.0018, φ = 0.40, stochastically significant after the Bonferroni multiple-comparison correction], but not in the control group [chi-square test, X^2^(1) = 5.19, *p* = 0.311. φ = 0.15]. We also found that the ratio of “No werewolf” votes after a round without a werewolf was significantly higher in the *Hugvie* than in the control group [chi-square test, X^2^(1) = 7.58, *p* = 0.0018, φ = 0.40, stochastically significant after the Bonferroni multiple-comparison correction]; however, there was no significant difference in the ratio of “No werewolf” votes after a round with a werewolf between two groups [chi-square test, X^2^(1) = 5.19, *p* = 0.311. φ = 0.15]. These results suggest that participants using *Hugvie* tended to have a biased belief that no werewolf existed in the current round after a round without a werewolf, although the identities of participants in the current round were independent from those in the previous round (**Figure 6**).

The winning percentage for the villagers was 37.5% in the *Hugvie* group and 62.5% in the control group. There was no significant difference in winning percentages between the two groups [chi-square test, X^2^(1) = 1.41, *p* = 0.32. φ = 0.28].

**FIGURE 7:**
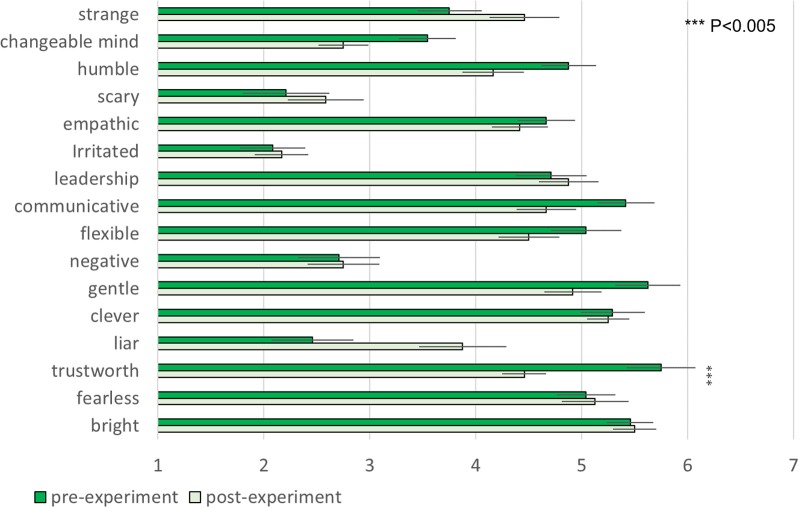
Changes in participants’ impressions of other players in the *Hugvie* group.

**FIGURE 8:**
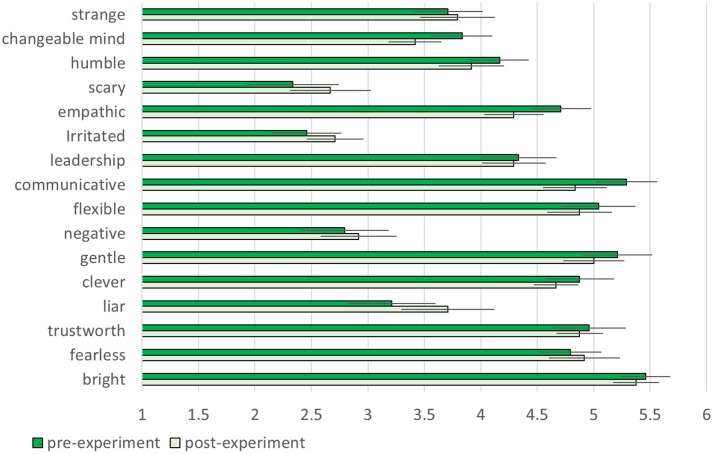
Changes in participants’ impressions of other players in the control group.

There was no significant difference of participants’ impressions of other players between two groups in any items of pre-experiment questionnaire. This means that the first impression of other players were controlled between two groups. The comparison of pre- and post-experiment showed that only the *Hugvie* group displayed a significant decrease in the post-test score on trustworthiness [**Figures 7, 8** paired *t*-test, *t*(11) = 4.08, *p* = 0.0018, *d* = 1.49, stochastically significant after the Bonferroni multiple-comparison correction].

## Discussion

The present research sought to verify the hypothesis that using *Hugvie*, a remote communication device combining sound with tactile sensations, maintains the level of trust that a user feels toward his/her conversation partner in an ambiguous situation. We used a game called *Werewolf* with modified rules for the purpose of this study, creating the possibility that no werewolf existed among the players. When playing the game remotely, the likelihood of players guessing that others were not werewolves was maintained when using *Hugvie* as opposed to using a basic sound-based communication device. These results support our hypothesis that using *Hugvie* maintains the level of trust of a user feels toward his/her remote conversation partner. We believe that using *Werewolf*, a game based on free-flowing conversation, allowed us to obtain such a result.

However, our study also showed that using *Hugvie* did not increase the villagers’ winning rate. In fact, the winning rate of *Hugvie* users was lower than that of the control group, suggesting that the level of trust that *Hugvie* users felt toward the other players was not based on reasoning. Research in social psychology has developed a scale to measure individuals’ general trust toward others (Yamagishi et al., 2015). The kind of trust that increased by using *Hugvie* may be general, rather than specific. The results of the present study also demonstrated that participants in the *Hugvie* group felt a significantly lower level of trust toward the other players after the experiment, which is presumably due to the villagers’ low winning rate. In other words, these participants may have regretted having trusted the others after seeing that it did not lead to the desired outcome, which subsequently lowered their level of trust. It can therefore be surmised that *Hugvie*’s positive effect on the level of trust toward others in remote communication may backfire if the situation becomes less agreeable, leading to users feeling regretful when remembering previous conversations. Consequently, this device may be more useful in natural daily conversations in which neither person has a vested interest. In a negative sense, our results might suggest that using *Hugvie* in remote communication leads to decreased thinking and might generate blind obedience to other persons. For example, in Japan, frauds targeting elderly people using mobile phones become a social problem. Thus, it is important to consider from an ethical viewpoint how *Hugvie*-like remote communication devices may alter our communication styles.

*Hugvie* is a remote communication device that provides the user with the sensation of a hug in addition to audio communication. However, the following question remains: to whom does the user attribute this sensation? For example, if two lovers are in conversation using this device, it is likely that they imagine each other as the one they are hugging, although the hugging sensation is not actually transmitted from their partner via *Hugvie*. In our experiment, however, the conversations involved three individuals; therefore, it was more difficult to attribute the hug sensation to a specific individual. The trust obtained through *Hugvie* may represent a generalized feeling caused by the physiological sensation of a hug during remote communication, rather than it being attributed to a specific individual. Research in embodied cognitive science suggests that the state of a person’s body significantly influences the way in which he/she views the outside world (Ballard et al., 1997; Ybarra et al., 2000; Tschacher and Bergomi, 2015). The results of this study suggest that the physical posture of hugging along with the sensation of being hugged might influence feelings toward remote conversation partners without necessarily attributing them to a specific individual. This might be likened to having a remote conversation with someone while holding a baby, a family pet, or a security blanket. Of course, this interpretation is too speculative at present and some researchers may doubt the effects of embodiment cognition (Ranehill et al., 2015). Hence, further detailed studies need to be conducted to reveal how *Hugvie* affects trust levels.

In this study, we must be careful to over-generalize the results, because sample sizes were not so large. Especially, many previous studies have reported that both gender and social relationships (e.g., friends or strangers) strongly affect the level of trust toward others (Croson and Buchan, 1999). In the current study we could not find a salient difference between male and female participants, with our preliminary analysis suggesting that no significant differences in the ratio of “No werewolf” votes existed between female and male participants. However, the sample size in the current study was too small to confirm a gender difference, although gender and social relationship proportions were matched between the *Hugvie* and control groups. Future research is needed to investigate how gender and relationships influence the effect of *Hugvie* in remote communication.

Finally, we would like to emphasize that using conversation games such as *Werewolf* is useful to verify the function and validity of remote communication systems, as they are enjoyable for participants in comparison with conventional experimental tasks and enable the analysis of natural utterances and emotional expressions generated while playing. Recently, there are many communication devices using various communication channels, such as tactile stimulation, smell, light, text characters, and emotional icons. Conversation games might provide an experimental paradigm to uniformly investigate how these various devices alter our social attitudes and emotions during remote communication.

## Author Contributions

HT, MB, HO, JN, HS, and HI designed the research. HT, MB, JN, and HS performed the research. HT and HS wrote this paper.

## Conflict of Interest Statement

The authors declare that the research was conducted in the absence of any commercial or financial relationships that could be construed as a potential conflict of interest.
